# Efficacy of budesonide suspension in the treatment of lobar pneumonia by fiberoptic bronchoscopic alveolar lavage

**DOI:** 10.3389/fmed.2025.1598234

**Published:** 2025-06-25

**Authors:** Xin Ji, Hongjie Fu, Fang Zhang, Na Chen, Xiugui Xing, Jinghua Ji, Tian Zhou, Xiangqin Song

**Affiliations:** ^1^Department of Pediatric Respiratory, Binzhou Medical University Hospital, Binzhou, China; ^2^Department of Nursing, Binzhou Medical University Hospital, Binzhou, China; ^3^Department of Office of Fashion Construction, Binzhou Medical University Hospital, Binzhou, China

**Keywords:** budesonide, lobar pneumonia, fiberoptic bronchoscopy, alveolar lavage, immune function

## Abstract

**Objective:**

To probe the efficacy of budesonide suspension in treating lobar pneumonia by fiberoptic bronchoscopic alveolar lavage.

**Methods:**

A total of 176 preschool and school-age children with lobar pneumonia in the pediatric respiratory department of Binzhou Medical University Hospital from December 2020 to December 2021 were selected and divided into observation group (OG, *n* = 88) and control group (CG, *n* = 88) based on the wishes of their families. Children in both groups were given azithromycin sequential treatment. The CG was given fiberoptic bronchoscope alveolar lavage with sodium chloride injection, and the OG was treated with fiberoptic bronchoscope alveolar lavage with sodium chloride injection + inhalation of budesonide suspension. The treatment, inflammatory factor levels, immune function, lung function indicators, adverse reactions and clinical efficacy were compared between both groups.

**Results:**

The total effective rate of the OG was 90.91%, which was better than 81.82% of the CG (*χ*^2^ = 3.095, *p* < 0.05). After treatment, the serum IL-6, CRP and ESR levels declined, and IL-2 level was elevated in the OG relative to the CG (*p* < 0.05). The levels of IgM, IgA, IgG, FVC, FEV1, PEF, FEF25, FEF50, FEF75 and FEF25-75 in the OG were enhanced compared to the CG (*p* < 0.05). The antipyrexia time, the disappearance time of lung shadow, the improvement time of cough and expectoration, and the disappearance time of lung rales in the OG were shorter compared to the CG (*p* < 0.05).

**Conclusion:**

Budesonide combined with fiberoptic bronchoscopy lavage in treating lobar pneumonia in children with *Mycoplasma pneumoniae* is effective in enhancing the therapeutic effect, relieving the clinical symptoms and signs of children faster, significantly reducing the inflammatory response, and significantly improving lung function. It is safe, effective and has no adverse reactions.

## Introduction

Lobar pneumonia is a type of community-acquired pneumonia, which is an acute pulmonary parenchymal inflammatory change characterized by diffuse fibrin exudation in the alveoli. The lesions of patients are initially confined to the alveoli of one or more lung lobes, and rapidly spread to lung segments and lobes (1, 2). According to statistics, lobar pneumonia accounts for 28–34% of the causes of death in children under five years old worldwide, which has brought a heavy burden to the global medical cause (3). At present, lobar pneumonia is no longer mainly caused by *Streptococcus pneumoniae*, and mixed infections such as viruses, bacteria, fungi and mycoplasma are also increasing year by year, especially lobar pneumonia caused by mycoplasma infection (4–6). Despite the similarities between children and adults with lobar pneumonia, evidence from adult studies cannot be transferred with certainty to treatment programs for children. Because colonization of the pharynx and trachea differs in young children and adults, pharmacokinetics, pathogenesis, and pathogen types varyn (7–9). Therefore, research on the treatment of lobar pneumonia in children is very necessary.

Budesonide suspension is still one of the main drugs for the treatment of lobar pneumonia in children. It has a highly effective local anti-inflammatory effect, which can promote the stabilization of endothelial cells and smooth muscle cells along the lysosomal membrane, inhibit immune response, reduce antibody synthesis, and lessen the release and activity of allergic active mediators such as histamine (10, 11). It is an important method for treating pneumonia, but the children are younger and have rejection to atomization inhalation. Fiberoptic bronchoscopy is a new technology developed on the basis of bronchoscopy that has been used in emergency, respiratory system, digestive system and other clinical treatments and has an irreplaceable position in medical experimental research (12). Fiberoptic bronchoscope lavage can dilute or even eliminate the sputum plug and inject medications into the trachea to fundamentally treat the disease because children with lobar pneumonia have a considerable volume of sputum in the trachea, and the sputum plug restricts the tracheal aperture. The majority of medical professionals think that using a fiberoptic bronchoscope lavage can improve how well budesonide is applied to treat lobar pneumonia (13). It offers unmatched advantages over traditional diagnostic and therapeutic methods, making it an essential diagnostic and therapeutic method for children with *Mycoplasma pneumoniae* pneumonia (MPP) (14).

In this study, 176 children diagnosed with lobar pneumonia in our hospital from December 2020 to December 2021 were treated with budesonide suspension combined with fiberoptic bronchoscopy lavage. Our study investigated the clinical efficacy of this combination therapy regimen in children with lobar pneumonia and found that, compared with traditional treatment, the combination therapy reduced lung inflammation and enhanced the treatment effect. It also provides a clinical reference for the treatment of lobar pneumonia in children.

## Methods

A total of 176 preschool and school-age children with lobar pneumonia in the pediatric respiratory department of Binzhou Medical University Hospital from December 2020 to December 2021 were selected. As shown in Table 1, the enrolled children were divided into observation group (OG, *n* = 88) and control group (CG, *n* = 88) according to the wishes of their families. There were 48 boys and 40 girls in the OG. The mean age was (6.12 ± 1.34) years (range 3–14 years). The mean course of disease was (5.94 ± 1.35) days (range 1–10 days). There were 50 boys and 38 girls in the CG. The mean age was (6.20 ± 1.12) years (range from 2 to 14 years). The mean course of disease was (6.10 ± 1.09) days (range 2–14 days). There were no significant differences in gender, age, and course of disease between both groups (*p* > 0.05). This study was reviewed and approved by the Medical Ethics Committee of our hospital.

**Table 1:** Comparison of general clinical data.

Characteristics	Observation group (*n* = 88)	Control group (*n* = 88)	*p* value
Male	48 (45.45%)	50 (56.82%)	0.7615
Age (months)	6.12 ± 1.34 (3–14)	6.20 ± 1.12 (2–14)	0.6679
Course of disease (days)	5.94 ± 1.35 (1–10)	6.10 ± 1.09 (2–14)	0.3882

### Ethical approval

This study was approved by the Ethics Professional Committee of Binzhou Medical College (No. BY2020KJ41).

### Inclusion criteria

- All patients met the diagnostic criteria of lobar pneumonia in the 8th edition of Zhu Fu Tang Practical Pediatrics.
- Pulmonary lesions were confirmed by X-ray examination and computer tomography.
- Accompanied by cough, fever and dyspnea.
- Aged from 3 to 14 years old.
- The lung imaging changes were all in one lobe.

### Exclusion criteria

- Patients with special infection such as fungal infection and tuberculosis infection.
- Patients with primary diseases of the heart, liver, kidney as well as hematopoietic system.
- Patients with respiratory failure, heart failure and other critical illness.
- Iatrogenic immunodeficiency caused by long-term use of immunosuppressants, cytotoxic drugs, budesonide, radiotherapy, etc.
- Long-term use of antibiotics;
- With immunodeficiency or congenital defects;
- Patients who were allergic to the drugs used in the study or whose instruments were not good enough.

Children in both groups were given azithromycin sequential treatment, azithromycin for injection [Manufacturer: Northeast Pharmaceutical Group Shenyang First Pharmaceutical Co., LTD., Approval number: Chinese Medicine approval number: H20000426, Specifications: Azithromycin 0.25 g (250,000 units)] 10 mg/kg was given intravenously once a day for five consecutive days and then stopped for two days. When the condition was stable, azithromycin dry suspension [manufacturer: Pfizer Pharmaceutical Co., LTD., Approval number: Chinese Medicine approval number H10960112, Specifications: 0.1 g (C38H72N2O12)], 10 mg/kg dissolved in an appropriate amount of water, was given orally, once a day, for three to five days.

Based on azithromycin sequential treatment, the CG was given fiberoptic bronchoscope alveolar lavage on day 2 after admission. Before fiberoptic bronchoscope alveolar lavage, children need to be fasted for 4 h to prevent aspiration and reduce the risk of gastric contents entering the airway. Electrocardiogram monitoring and blood oxygen monitoring are also needed before treatment to ensure the safety of children during the treatment. Lidocaine and chlorhexidine aerosol (Jiangsu Tianji Pharmaceutical Co. LTD., Chinese medicine approval number H32026131) were given in the nasal cavity and pharynx 30 min before the operation. The children were supine during the operation, and the front end of the bronchus fibrosus was fully lubricated with gel before the operation. The soft bronchoscope was slowly inserted into the nasal cavity of the child, into the bronchus through the nasal cavity, the bronchus and alveoli were observed, and then 37°C sodium chloride injection (Shandong Lukang Chenxin Pharmaceutical Co. LTD., Chinese medicine approval number H20056758) was injected through the tube to fully lavage. A negative pressure suction of 80–120 mmHg was used to suck out the mucus plug. If the mucus plug was seriously blocked and it was difficult to suck out by lavage, the biopsy forceps was used to suck out again, and then the bronchoscopy was removed. Vital signs, including respiratory rate, heart rate, and blood oxygen saturation, should be closely observed during lavage to ensure the safety of children.

Based on azithromycin sequential treatment and fiberoptic bronchoscope alveolar lavage, the OG was treated with inhalation of budesonide suspension (AstraZeneca Pty Ltd., Registration number: H20140475, Specification: 2 mL: 1 mg) inhalation therapy, 0.5–1 mg/time, 2 times/d, treatment for seven days.

### Observation indicators

(1) The antipyretic time, lung shadow absorption time, cough relief time, expectoration improvement time, lung rales disappearance time were observed in the two groups (2). Inflammatory response: 3 mL peripheral venous blood was gathered before and after treatment, centrifuged at 3000 r/min for five minutes, and the supernatant was gathered. Refer to the instructions for enzyme-linked immunosorbent assay (ELISA) kits (Invitrogen, United States) for serum interleukin-2 (IL-2) and interleukin-6 (IL-6). C reactive protein (CRP), erythrocyte sedimentation rate (ESR) as well as other inflammatory response related indicators (3). Pulmonary function: the pulmonary function of the children was detected by Micro Po Box6 pulmonary function detector provided by Germany Bairui Company before and after treatment. (a) Airway ventilation function: forced vital capacity (FVC), forced expiratory volume in one second (forced expiratory volume in one second, FEV1), peak expiratory flow rate (PEF) and 25% forced expiratory flow rate (FEF25); (b) Small airway ventilation function: forced vital capacity at 50% (FEF50), forced vital capacity at 75% (FEF75), maximum mid-expiratory flow (FEF25-75) (4). Adverse reactions: during the treatment, the occurrence of adverse reactions was closely monitored and recorded (5). Indicators of immune function: fasting venous blood samples were gathered before and after treatment. The levels of im-munoglobulin M (IgM), immunoglobulin A (IgA) and immunoglobulin G (IgG) were measured by ELISA (6). The clinical efficacy was compared between the two groups. The criteria were as follows: all clinical symptoms disappeared as marked effect; the clinical symptoms were reduced to effective. No improvement in clinical symptoms was considered ineffective. Total efficiency = obvious efficiency + efficiency.

### Statistical analysis

Use GraphPad Prism 8.0.1 to represent graphs as mean ± standard deviation (SD). Categorical variables are expressed as the number of cases (percentage) [n (%)], and component comparisons are determined by chi-square test. Statistical analysis of the results was performed by the student’s t-test (two groups) or one-way ANOVA (multiple groups). The difference is considered statistically significant when the *p*-value is less than 0.05.

## Results

**Figure 1:**
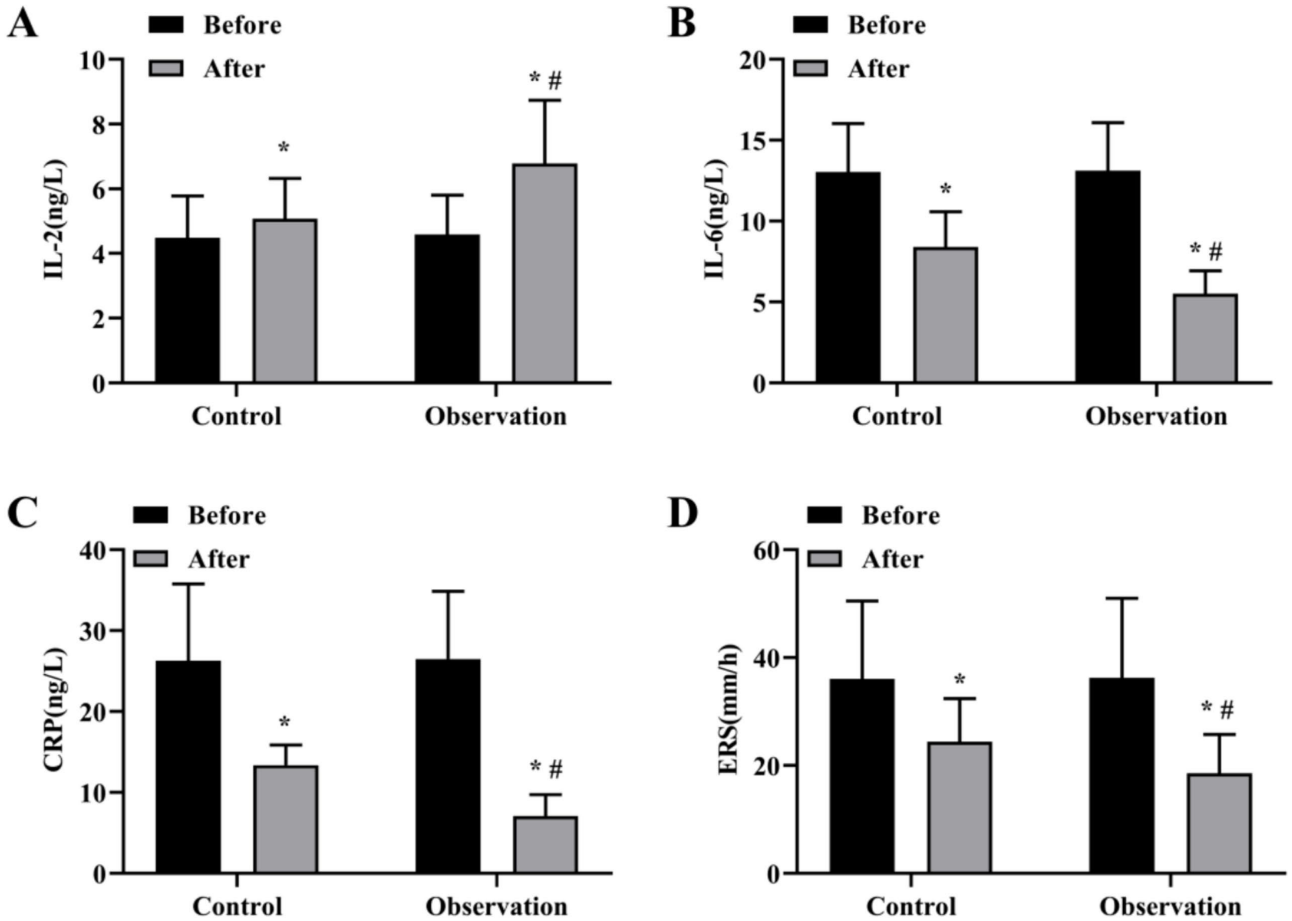
Levels of inflammatory factors before and after treatment in both groups. Serum levels of IL-2 **(A)**, IL-6 **(B)**, CRP **(C)** and ESR **(D)** content in OG and CG groups. *p < 0.05, compared with before treatment; #*p* < 0.05, compared with control group.

The total effective rate of the OG was 90.91%, which was better than 81.82% of the CG (*χ*^2^ = 3.095, *p* < 0.05), as displayed in Table 2. The levels of inflammatory factors in the two groups before and after treatment were detected. The results showed that the serum levels of IL-6, CRP and ESR in the OG group were lower than those in the CG group (Figures 1B–D), and the serum levels of IL-2 in the OG group were significantly higher than those in the CG group (Figure 1A) (*p* < 0.05).

**Table 2:** Clinical efficacy of children in both groups.

Groups	Cases	Obvious effective (%)	Effective (%)	Ineffective (%)	Total effective rate (%)
Observation group	88	36 (40.91)	44 (50.00)	8 (9.09)	90.91
Control group	88	20 (22.73)	52 (59.09)	16 (18.18)	81.82
*χ* ^2^		3.095			
*P*		<0.05			

**Figure 2:**
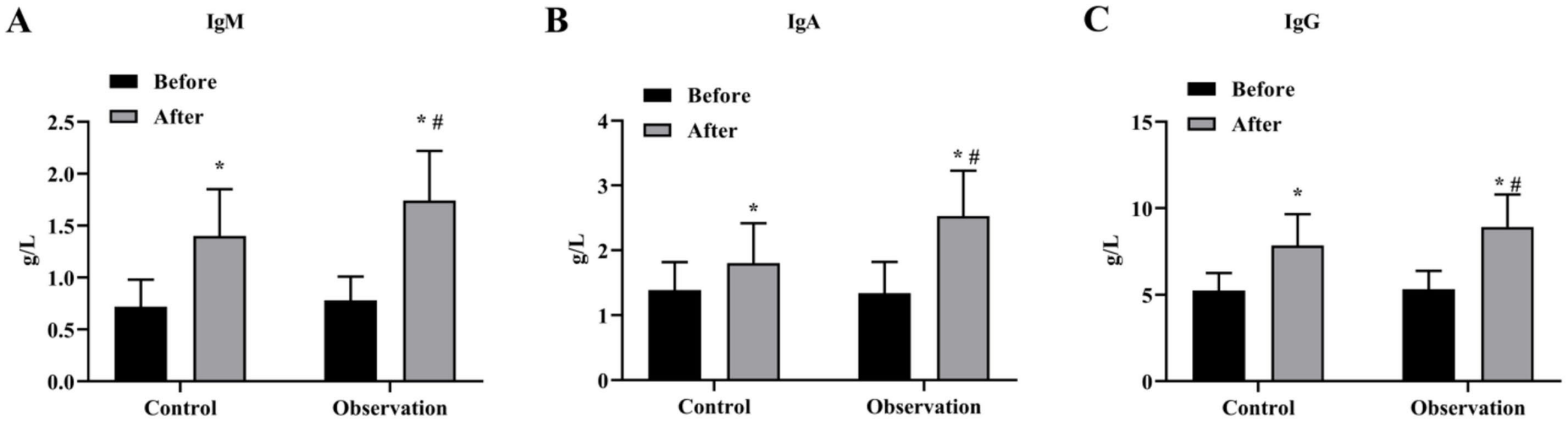
Immune function indexes of the two groups before and after treatment. Serum levels of IgM **(A)**, IgA **(B)** and IgG **(C)** content in OG and CG groups. *p < 0.05, compared with before treatment; #*p* < 0.05, compared with control group.

The results of ELISA showed that there was no significant difference in the levels of immune function indicators IgM, IgA and IgG before treatment (*p* > 0.05), and the immune function of the two groups was higher than that before treatment (*p* < 0.05) (Figures 2A–C). In addition, the levels of IgM, IgA and IgG in the OG group were higher than those in the CG group significantly (*p* < 0.05) (Figures 2A–C).

**Figure 3:**
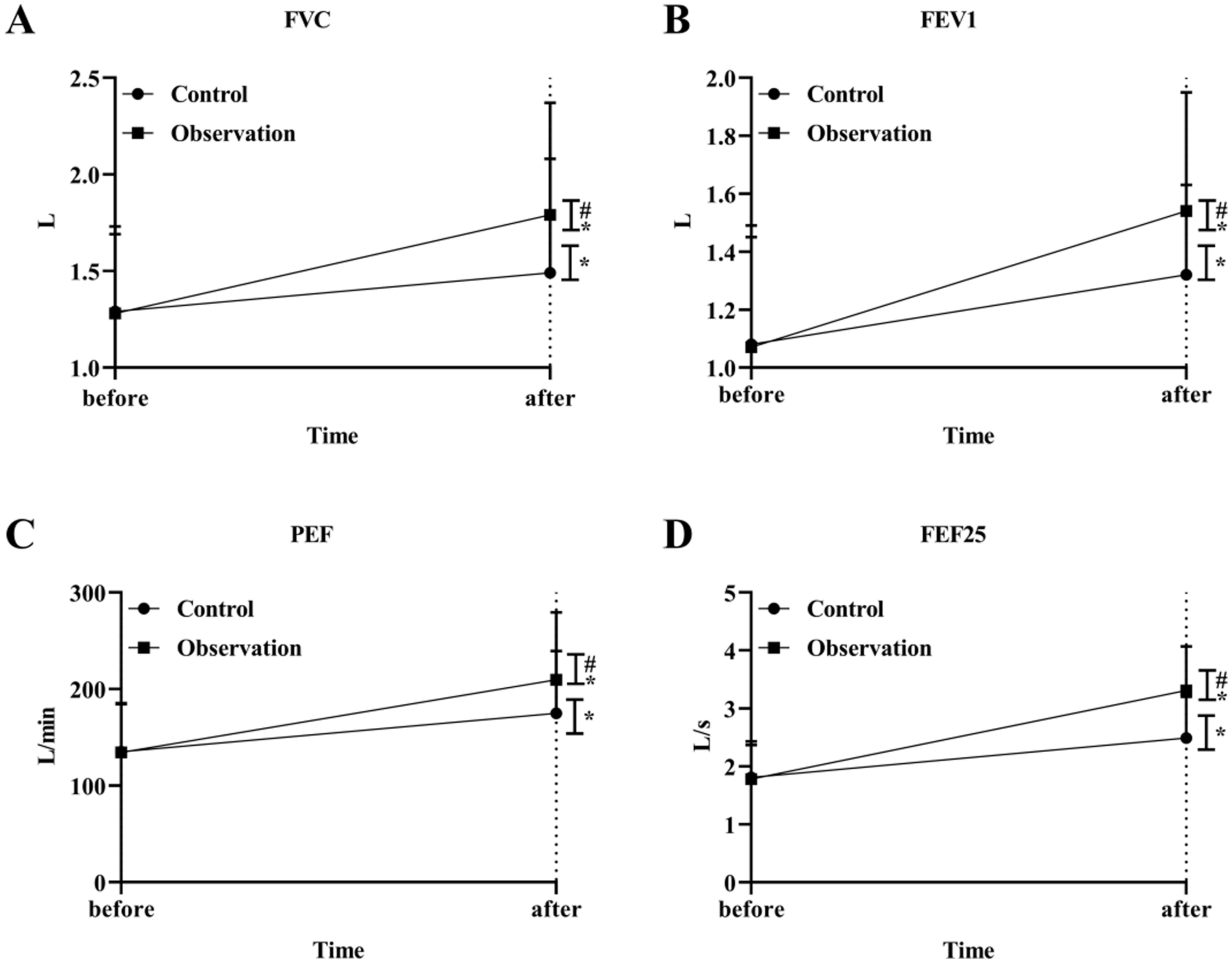
Big airway ventilation function in the two groups. The level of airway ventilation function index FVC **(A)**, FEV1 **(B)**, PEF **(C)** and FEF25 **(D)** in OG and CG groups. *p < 0.05, compared with before treatment; #*p* < 0.05, compared with control group.

**Figure 4:**
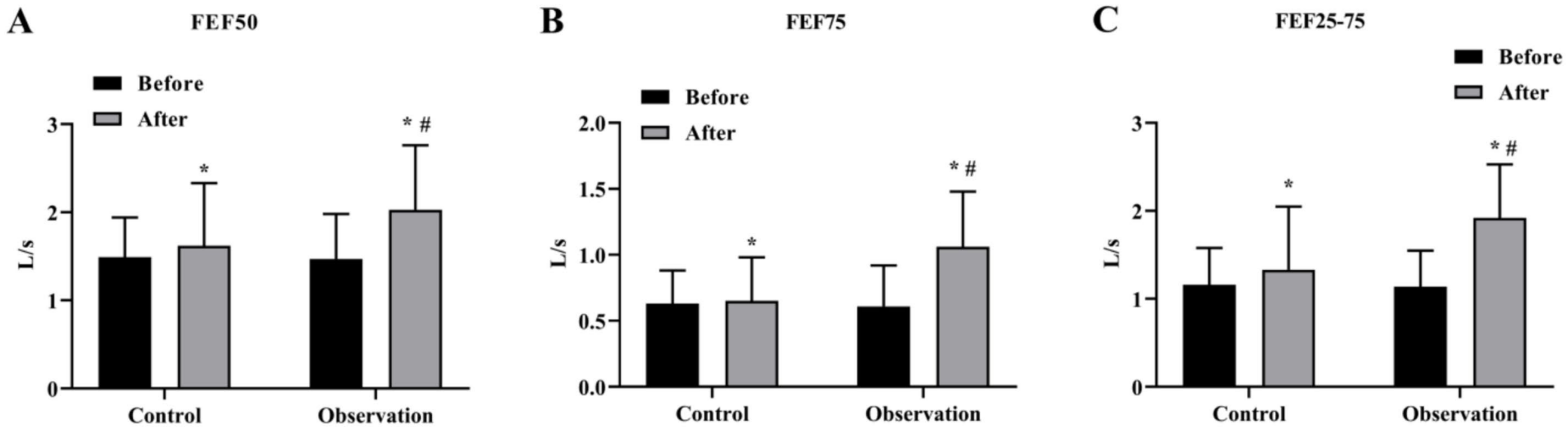
Small airway ventilation function in the two groups. The level of small airway ventilation function index FEF50 **(A)**, FEF75 **(B)**, and FEF25-75 **(C)** in OG and CG groups. *p < 0.05, compared with before treatment; #*p* < 0.05, compared with control group.

We then evaluated the ventilation function of the large airways. The results showed that the levels of FVC, FEV1, PEF and FEF25 were increased after treatment in both groups. The levels of FVC, FEV1, PEF and FEF25 in the OG group were better than those in the CG group after treatment significantly (*p* < 0.05) (Figures 3A–D). In addition, the results of small airway ventilation function assessment also showed that the levels of FEF50, FEF75, and FEF25-75 were improved after treatment, and the OG group had a more significant effect than the CG group (*p* < 0.05) (Figures 4A–C).

The antifever time, the disappearance time of lung shadow, the improvement time of cough and expectoration, and the disappearance time of lung rales in the OG were shorter compared to the CG (*p* < 0.05), as revealed in Table 3. The incidence of adverse reactions in the OG (9.09%) was lower compared to the CG (11.36%), but there was no statistically significant difference (*p* > 0.05), as displayed in Table 4.

**Table 3:** Treatment conditions of the two groups.

Groups	Cases	Defervescence time	Disappearance time of cough	Expectoration improvement time	Disappearance time of pulmonary rales	Disappearance time of the lung shadow
Observation group	88	3.21 ± 1.26	7. 19 ± 1. 54	4. 49 ± 1. 32	3. 21 ± 1. 09	2. 21 ± 0. 95
Control group	88	5.33 ± 2.17	3. 32 ± 1. 51	9. 74 ± 2. 47	7. 26 ± 1. 49	4. 72 ± 1. 42
*χ* ^2^		4. 926	3. 628	5. 108	8. 114	4. 919
*P*		<0.05	<0.05	<0.05	<0.05	<0.05

**Table 4:** Incidence of adverse reactions in both groups.

Groups	Cases	Transient hypoxemia (%)	Polypnea (%)	Worsening of cough (%)	Fever (%)	Nausea and vomiting (%)	Incidence of complications (%)
Control group	88	3 (3.41)	2 (2.27)	1 (1.14)	2 (2.27)	2 (2.27)	10 (11.36)
Observation group	88	2 (2.27)	1 (1.14)	1 (1.14)	2 (2.27)	2 (2.27)	8 (9.09)
*χ* ^2^		0.901					
*P*		>0.05					

## Discussion

The body stimulates immunocompetent cells to produce inflammatory cytokines after *Mycoplasma pneumoniae* infection, which aggravates pulmonary inflammation and induces various extrapulmonary systemic complications (15, 16). Studies have shown that the use of budesonide in the treatment of pneumonia can effectively decline the exudation of pulmonary inflammation in children, accelerate the relief of bronchospasm, and help improve the vascular permeability and microcirculation of children (17, 18). However, children with lobar pneumonia have severe bronchial mucosal congestion, varying degrees of edema and exudation, and many viscous secretions. After a period of budesonide treatment, the viscous secretions still cannot be completely discharged, and the drug concentration in the lesion site cannot achieve the ideal therapeutic effect (19). Therefore, budesonide is used in combination with other treatments to optimize the treatment of lobar pneumonia and improve clinical efficacy.

In this study, budesonide suspension combined with fiberoptic bronchoscopic lavage therapy significantly improved the outcome of children with lobar pneumonia. Previous studies have shown that fiberoptic bronchoscopy lavage technology can repeatedly wash the bronchus in the lesion site, which on the one hand can send the bronchial specimens in the lesion site for examination, and on the other hand, it can also relieve airway obstruction and help discharge inflammatory secretions, which is conducive to shortening the course of treatment (20). If necessary, the operator can also thoroughly clean the granulation tissue and mucus plug by direct vision, and local injection of antibiotics in the lesion site (21, 22). A study of 54 children with refractory mycoplasma pneumonia treated with budesonide combined with fiberoptic bronchoscopy lavage, it was found that compared with the control group, the combination treatment group significantly improved the clinical symptoms of children, enhanced ventilation function, and reduced the level of inflammatory mediators (10). The same results were also shown in this study, with the antifever time, the disappearance time of lung shadow, the improvement time of cough and expectoration, and the disappearance time of lung rales in OG patients were shorter than those of CG and there was no significant difference in the incidence of adverse reactions between both groups, suggesting that the combined regimen was safe. The above results show that budesonide combined with fiberoptic bronchoscope lavage in the treatment of lobar pneumonia can effectively improve the inflammatory response and pulmonary function of children, which is conducive to shortening the course of treatment and enhancing the efficacy.

To further investigate the important role that combination therapy plays in immune function, the present study examined serum levels of inflammatory factors (IL-6 and IL-2), antibodies (IgM, IgA, and IgG), as well as CRP and ESR levels in children with lobar pneumonia. In addition, IL-2 is essential for the immune response and helps to regulate the immune response by activating regulatory T lymphocytes (23, 24). IL-6 is recognized as a central mediator of the cytokine cascade and is a well-known pro-inflammatory cytokine (25). In addition, after infection with *Mycoplasma pneumoniae*, the body produces appropriate antibodies to form immune complexes to fight the infection (26). Studies have shown that T-helper 17 (Th17) cells, a subset of CD4^+^ T helper cells, play an important role in host defense and clearance of bacterial and fungal pathogens in the lung (27). Th17 cells differentiate in the context of proinflammatory cytokines and secrete cytokines such as IL-17A, IL-17F, and IL-22, which also have proinflammatory properties (28). It has been reported that budesonide can modulate the balance of regulatory T cells (Tregs)/Th17 cells in the treatment of asthma (29). Human leukocyte antigen-G (HLA-G), a polymorphic non-classical HLA (HLA-Ib) with immune-regulatory properties in infectious diseases (30). The membrane-bound and soluble forms of human leukocyte antigen-G (HLA-G) molecules play a central role in the regulation of immune responses (31). Based on the current results, we found that the combination therapy in the OG group suppressed the inflammatory response faster and enhanced the immune function. In children with lobar pneumonia, budesonide combined with flexible bronchoscopic alveolar lavage therapy can safely and effectively alleviate the clinical symptoms of children and provide an effective reference for future clinical work.

### Limitations

There are still some limitations to this study. The number of children with lobar pneumonia included in this study is limited. In addition, as the study was conducted in a single center, there may be bias, which may limit the applicability of the results to a wider population and we will continue to expand the clinical sample size in the future to provide more rigorous and credible clinical data.

## Conclusion

In treating children with *Mycoplasma pneumoniae* lobar pneumonia, budesonide in combination with fiberoptic bronchoscopy lavage assists to improve the therapeutic effect, reduce the inflammatory response, expedite the resolution of clinical symptoms and signs, shorten the duration of treatment, and improve lung function. It is also safe and effective with no adverse reactions.

## Data Availability

The original contributions presented in the study are included in the article/supplementary material, further inquiries can be directed to the corresponding author.
